# Predicting Consumer Purchase Intention for Pre-Prepared Meals Based on Random Forest and Explainable AI (SHAP): A Study in Jilin Province, China

**DOI:** 10.3390/foods15050896

**Published:** 2026-03-05

**Authors:** Xiaodan Qi, Hongyan Zhao, Xihe Yu

**Affiliations:** School of Public Health, Jilin University, Changchun 130021, China; xiaodan980618@gmail.com (X.Q.);

**Keywords:** pre-prepared meals, consumer purchase intention, machine learning, random forest, SHAP interpretability, Bayesian optimization

## Abstract

The pre-prepared meal industry is a vital engine for food sector upgrading in China. This study investigates the key drivers of consumer purchasing decisions and identifies strategic pathways to support high-quality industry development. Grounded in behavioral decision theory and the stimulus–organism–response framework, we propose two central research questions: (1) What are the dominant determinants of consumer purchase intention for pre-prepared meals? and (2) How do these determinants interact in nonlinear and asymmetric ways to shape final decisions? To address these questions, we analyzed 805 valid questionnaires collected in Jilin Province using an integrated machine learning framework. Data quality and validity were ensured through baseline balance tests, and sample imbalance was corrected using the SMOTE–Tomek algorithm. Six models, including Random Forest (RF) and XGBoost, were optimized via Gaussian process-based Bayesian optimization. The RF model achieved optimal performance on the test set, with an F1 score of 0.907, an AUC of 0.928, and a prediction accuracy of 0.876. To enhance model interpretability, Mean Decrease Impurity (MDI) was integrated with the SHAP framework. Our findings reveal that: (1) purchase decisions are predominantly willingness-driven, with behavioral tendency—especially recommendation willingness—accounting for over 72% of predictive importance; (2) rational considerations, such as convenience and channel accessibility, serve as foundational enablers; and (3) recommendation willingness exhibits a significant S-shaped nonlinear threshold, where a shift to “relatively willing” marks a critical marketing intervention window. SHAP force plot analysis further uncovers an asymmetric decision logic: high willingness can compensate for perceived product shortcomings, whereas the absence of core intention functions as a non-compensatory barrier. Theoretically, these findings synthesize machine learning outputs with classical behavioral models (e.g., the Theory of Planned Behavior and Prospect Theory) by empirically quantifying bounded rationality and nonlinear activation mechanisms. These findings suggest that enterprises should transition from traffic-centric to retention-oriented strategies by leveraging word-of-mouth and proximity-based channels. Moreover, establishing a collaborative governance system is essential to mitigate risk perception and ensure long-term industry prosperity.

## 1. Introduction

Pre-prepared meals are prepackaged dishes made from one or more edible agricultural products and products derived from them, which can only be consumed after industrial pre-processing, heating, or cooking. They do not include staple foods. There are various types of pre-prepared meals, and the raw materials are relatively complex. There are high requirements regarding the freshness of ingredients and storage conditions. This category includes pre-prepared meals, frozen foods, meal packs, and canned and vacuum-sealed products. In recent years, pre-prepared food has become increasingly popular in China [1]. This expansion may be driven by several key factors, including rapid urbanization, the fast pace of modern lifestyles, and the continuous advancement of food preservation and preparation technologies. By 2025, China is expected to contribute approximately 170 billion US dollars, accounting for about a quarter of the total market revenue [2].

As a key path for the transformation and upgrading of China’s food industry, the ready-to-eat meal industry is becoming an increasingly important force leading the reform of the agricultural supply side and new trends in food consumption. Jilin Province, as a major agricultural resource province, enjoys unique resource endowments and geographical advantages in terms of developing the pre-prepared catering industry. The pre-prepared meal industry chain involves links such as agricultural product production, pre-prepared meal processing, transportation, and sales. Its upstream is the supply of raw materials, the midstream is the production and processing of pre-prepared meals, and the downstream is the consumer market [3]. The localization development of this industry not only promotes more intensive processing of agricultural products, extends the agricultural industrial chain, and increases the added value of agriculture but also holds significant strategic importance for promoting the revitalization of rural industries, optimizing the food supply system in urban and rural areas, and meeting residents’ increasingly high consumption quality demands [4]. The aim of food safety is to ensure that food does not pose a risk to consumers’ health during its production, processing, storage, transportation, and sale. Safety, especially with respect to food, is the top priority. High-profile food safety incidents, such as the melamine-tainted milk powder scandal in 2008 and the recent exposure of counterfeit Thai fragrant rice during the 2024 CCTV 3·15 Gala, have seriously affected consumer confidence and public health. In the absence of complete guarantees of food safety, the rapid development of pre-prepared meals has raised concerns about health [5].

Studies show that pre-prepared meals usually contain high levels of sodium, saturated fat, and preservatives, all of which are associated with various health problems, such as hypertension, obesity, and cardiovascular diseases [6,7]. Therefore, in order to develop the pre-prepared meal industry rapidly and safely, the government has formulated food safety regulations and monitoring plans and conducts routine inspections of food enterprises, as although convenience foods have become a part of daily diets in developed countries [8,9,10], it is generally believed that pre-prepared meals have insufficient quality, taste, and nutrition. These concerns are exacerbated by the fact that the food is usually high in salt, which is unhealthy [11]. Therefore, during food inspection, inspectors need to verify whether these enterprises’ production complies with hygiene standards and food-handling procedures and whether they can cooperate with operators to identify risks and formulate practical solutions [12]. In the process of industrial expansion and market cultivation, consumers’ purchasing behavior constitutes the most direct driving force of market expansion. Its decision-making process is far from being a simple matter of price and preferences; it is embedded in complex socio-economic networks and individual psychological cognitive systems.

Consumers’ willingness to purchase pre-prepared food is essentially the result of the combined effect of multiple factors. From the micro perspective, i.e., at the individual level, it involves consumers’ emotional attitudes and subjective preferences towards the taste, convenience, and nutritional value of the product. From a meso-cognitive perspective, it includes rational assessments and risk perceptions regarding food safety, brand trust, and rational pricing. From a macro, social environment perspective, it is shaped by multiple external forces, such as traditional food culture, changes in family structure, the pace of urban life, media information dissemination, and the atmosphere of social trust. These factors interweave with each other, jointly forming a complex dynamic system that influences purchasing decisions. However, research in this field mostly focuses on impact analyses of a single dimension or limited variables, such as methods from industry and management perspectives like supply chain resilience [13] and regulatory laws [14], or focuses on the behavioral characteristics of consumers in specific regions. There are still no systematic analysis frameworks that integrate emotional, cognitive, and environmental factors, and there are even fewer studies that use modern data analysis techniques to comprehensively reveal the nonlinear interactions among these factors. One survey showed that 93% of American consumers believe that retailers comply with safety standards. They believe that in the event of a recall or an epidemic, retailers will immediately remove products from the shelves [15,16]. China’s pre-prepared meal industry can also draw on the management methods of the United States to make consumers feel more at ease when making purchases.

Notably, consumers’ perceptions of the risks of pre-prepared meals constitute a key obstacle in their decision-making process and are directly related to the issue of collaborative governance of food safety. From the process and supply chain management at the production end [17,18,19] to the traceability supervision at the circulation end [20] and strategic and performance management [13,14,15,16] and fiscal and tax planning [21] at the enterprise end, as well as macro industrial regulation and investment evaluation [22,23], a vast collaborative governance network has been formed. Risk perception theory [24] is often used to analyze consumers’ acceptance of emerging food technologies. In addition, the relationship between supply chain traceability and consumer trust [25], as well as research on the food market under conditions of information asymmetry [26], also provides theoretical references useful for understanding the trust challenges faced by the Chinese pre-prepared-food market.

Although food inspection plays a core role in protecting public health, there is still a clear lack of research on applying advanced machine learning, deep learning, and transformer-based modeling to this field, especially in terms of integrating explainability into methods that support transparency, accountability, and practitioner trust. Studies [27,28,29,30,31] have explored food safety and inspection issues from multiple perspectives, but they have largely overlooked the significant role of the explainability and transparency brought about by methods such as SHAP. Accordingly, based on an empirical investigation of the pre-prepared-food consumption market in Jilin Province, we aimed to construct a systematic and multi-dimensional analytical framework to bridge the gap between micro-consumption behavior and macro-industrial governance in existing research.

This study is based on first-hand questionnaire survey data and rigorously employed quantitative research methods. Following an assessment of basic data quality, the robustness of research tools was ensured through reliability and validity tests. Then, the key influencing factors were identified using feature-screening technology. On this basis, machine learning algorithms were introduced to build predictive models, capturing complex patterns and interaction effects that are difficult to reveal via traditional statistical methods. Finally, through an analysis of the model’s interpretability, the specific action paths and relative importance of each influencing factor were clarified. This complete analytical chain, stretching from data validation and feature mining to model construction and interpretation, can not only more comprehensively and accurately reveal the core driving forces behind consumption behavior regarding pre-prepared meals but also feed the micro-empirical evidence of consumer decision-making back to the collaborative governance system, composed of government supervision, industry self-discipline, technical support, and consumer participation. We aimed to provide solid empirical evidence and scientific decision-making references for building an evidence-based and dynamically responsive food safety governance model, thereby promoting the stable and long-term development of the pre-prepared meal industry while balancing high-quality growth and food safety assurance.

## 2. Materials and Methods

### 2.1. Questionnaire Design and Data Collection

Using a mature theoretical framework of consumer behavior, we designed a structured questionnaire designed to explore consumers’ purchase intentions in Jilin Province, China, with respect to pre-prepared meals and the influencing factors. The core part of the questionnaire contains ten psychological and behavioral constructs measured via a 5-point Likert scale (1 = “strongly disagree/Have no influence at all”, 5 = “strongly agree/have influence”): emotional attitude, cognitive attitude, subjective norms, perceived control, behavioral tendency, product attributes, convenience, risk and safety, price promotion, and channel access. The target variable “purchase intention” was derived from item E1 (“I am willing to purchase Pre-prepared meals in the next month “), and it is divided into “willing” (with a score of 4–5) and “unwilling” (with a score of 1–3). Meanwhile, the questionnaire also collected demographic information (gender, age, education level, occupation, monthly income, etc.) and past purchasing behavior data.

The data were collected through an online research platform, Wenjuanxing (Changsha Ranxing Information Technology Co., Ltd., Changsha, China; https://www.wjx.cn), from multiple locations in Jilin Province, such as Changchun, Jilin, and Siping. After the invalid answers were eliminated, a total of 805 valid questionnaires remained. This study adhered to academic ethics norms, and all participants provided informed consent.

### 2.2. Data Preprocessing and Reliability Analysis

This article is based on a consumer survey questionnaire of pre-prepared meals in Jilin Province. The original items were simplified into abbreviations of letters and numbers to facilitate subsequent analysis. A specific comparison table is shown in Supplementary Table S1. Meanwhile, the original data from the questionnaire contained text-based variables, which needed to be converted into numerical types for modeling. A table showing the encoding rules is given in Supplementary Table S2, all data preprocessing and statistical analyses were performed using Python software (version 3.9; Python Software Foundation, Wilmington, DE, USA).

Reliability is a core indicator for measuring the internal consistency of a scale, reflecting the measurement stability of questionnaire items in the same dimension. Cronbach’s Alpha coefficient is a classic method for evaluating the reliability of scales that can be used to test the internal consistency of the Likert Scale.

The Cronbach coefficient measures the reliability of a scale based on the “covariance between items”. Its core logic is that if all items in the scale measure the same latent construct (such as “emotional attitude”), there should be a significant positive correlation between the items. The higher the α coefficient, the stronger the internal consistency. Academically, it is generally believed that α < 0.6 indicates insufficient reliability, 0.6 ≤ α < 0.7 is acceptable, 0.7 ≤ α < 0.8 is good, and α ≥ 0.8 is excellent.

The Cronbach coefficient can be calculated using Equation (1):

(1) $$\alpha =\frac{k}{k-1}\left(1-\frac{\sum_{i=1}^{k}\sigma_{x_{i}}^{2}}{\sigma_{x_{total}}^{2}}\right)$$

Here, k represents the number of items in the scale dimension (for example, the dimension “Emotional Attitude” contains a total of 4 items from A1 to A4, with k = 4); $\sigma_{x_{i}}^{2}$ is the variance of the score for the i-th item; and $\sigma_{x_{total}}^{2}$ is the variance of the total score of all the items in the dimension. If there are missing values in the questionnaire items, they need to be filled in through methods such as determining the mode and mean, and then the variance can be calculated. For reverse scoring items, it is necessary to first reverse the scoring direction to ensure there is a positive correlation among the items.

### 2.3. Feature Screening and Dataset Construction

To identify the key predictor variables, the Pearson correlation coefficients between all candidate features and the binary target variable (purchase intention) were calculated. The Pearson correlation coefficient measures the degree of linear correlation between continuous variables, with values ranging from [−1, 1]. *r* > 0 indicates a positive correlation; *r* < 0 indicates a negative correlation, and the closer r is to 1, the stronger the correlation; *r* < 0.3 indicates a weak correlation; 0.3 ≤ *r* < 0.5 indicates moderate correlation; and *r* ≥ 0.5 indicates a strong correlation. The Pearson correlation coefficient is calculated using Equation (2):

(2) $$r=\frac{\sum_{i=1}^{n}\left(X_{i}-\bar{X})(Y_{i}-\bar{Y}\right)}{\sqrt{\sum_{i=1}^{n}{\left(X_{i}-\bar{X}\right)}^{2}\sqrt{\sum_{i=1}^{n}{\left(Y_{i}-\bar{Y}\right)}^{2}}}}$$

The features that had a correlation coefficient (*r*) > 0.2 and were deemed statistically significant (*p* < 0.05) were screened out for subsequent modeling, and ultimately 15 key features were obtained. The total dataset was divided into a training set (*n* = 644) and a test set (*n* = 161) in the classic 8:2 ratio by using the stratified random sampling method to ensure there was a consistent distribution of the target variable in the two groups. The unbiasedness of this division with respect to key demographic variables was verified through the baseline data balance test (chi-square test).

### 2.4. Category Imbalance Handling

Sample imbalance is a common problem in classification tasks (for example, in this article, the ratio of “unwilling to purchase” to “willing to purchase” samples is approximately 7:3). Although a single SMOTE oversampling can balance the quantity, it is likely to cause class overlap and introduce noise. Therefore, to prevent the model from being biased towards majority fatigue, we employed the SMOTE–Tomek hybrid resampling algorithm to process the training set data. The SMOTE–Tomek algorithm significantly improves the classification performance of the model by combining the dual mechanisms of “oversampling” and “undersampling” to clean the boundaries while generating samples.

The SMOTE–Tomek algorithm involves two core steps: Firstly, the SMOTE algorithm is used to synthesize new samples of the minority class, making the number of samples of each class balanced. Subsequently, the Tomek Links rule is utilized to identify and eliminate sample pairs in the feature space that are mutually nearest neighbors but of opposite categories (i.e., boundary noise), thereby constructing a clearer classification boundary.

### 2.5. Machine Learning Modeling and Hyperparameter Optimization

We systematically constructed and compared six classic machine learning classification models, namely, logistic regression, K-nearest neighbors, support vector machines, decision trees, random forests, and extreme gradient boosting trees (XGBoost) [32]. To overcome the limitations of traditional hyperparameter-tuning methods, we introduced the Bayesian optimization algorithm as the core method for optimizing the performance of each model and constructed an automated and highly efficient hyperparameter-tuning framework.

In this study, Bayesian optimization was applied to adjust the core parameters of each machine learning model, such as the number of decision trees, maximum tree depth, minimum number of split samples, regularization coefficients, and kernel function types. For the XGBoost model, hyperparameters including the learning rate, tree depth, and sub-sampling ratio were also automatically optimized. We set up a 80-round optimization process. Each iteration included updating the Gaussian process proxy model based on existing evaluation results and obtaining performance metrics on the validation set to gradually narrow down the search range. This targeted approach was particularly important in our multi-model comparison, as it ensured that each model could perform at its best under fair conditions, providing a reliable comparison basis for ultimately determining Random Forest to be the optimal model. Through this method, we systematically and reproducibly completed the parameter optimization of complex models, laying a solid technical foundation for the subsequent analysis.

### 2.6. Model Evaluation and Interpretability Analysis

We adopted a comprehensive model evaluation and interpretation strategy, aiming to ensure the reliability of the prediction results and reveal the decision-making logic behind them. Model performance was comprehensively evaluated on an independent test set, and a series of classic metrics were selected: accuracy measures the overall prediction accuracy rate; precision focuses on the accuracy of the model when predicting positive examples; recall rate measures a model’s ability to identify actual positive examples; the F1 score, as the harmonic mean of precision and recall, provides a robust assessment of imbalanced data; and the area under the receiver operating characteristic curve reflects the overall classification ability of the model at different thresholds. In-depth analysis of the confusion matrix further reveals the specific types of classification errors, providing key details for understanding the model’s behavior.

To address the “black box” nature of machine learning models and enhance the interpretability of the results, we constructed a dual-path interpretation framework. Firstly, we utilized the inherent interpretation mechanism of the model: for ensemble models based on decision trees such as random forest and XGBoost, we calculated feature importance based on the reduction in Gini impurity. Importance, I, can be estimated using Formula (3) as follows:

(3) $$Importance(j)=\sum_{t=1}^{T}\frac{\left|D_{t}\right|}{\left|D\right|}\Delta Impurity(t,j)$$

Here, D_t_ is the sample set of the t-th node, and Impurity(t, j) is the reduction in impurity of feature j when node t is split. This metric quantifies the global contribution of each feature to the overall prediction from within the model.

Next, a model-independent local explanation method is introduced, namely, a SHAP framework based on the Shapley value of cooperative game theory, to provide a consistent and individualized feature attribution explanation for each prediction. The core of the Shapley value is “fair distribution”: the difference between the predicted value of the sample and the baseline value (the average predicted value of all samples) is determined by the fair distribution of each feature. For sample x, the SHAP value of feature j is

(4) $$\phi_{j}(x)=\sum_{S\subseteq F\;\left\{j\right\}}\frac{\left|S\right|!(\left|F\right|-\left|S\right|-1)!}{\left|F\right|!}\left[f(S\cup \left\{j\right\})-f(S)\right]$$

By aggregating the SHAP values of all samples, a global feature importance ranking (summary graph) can be generated, and the nonlinear relationship between individual features and the model output can be revealed through a dependency graph. This dual verification system that combines endogenous measurement with external attribution not only enhances the robustness of the conclusions but also, more importantly, transforms the model’s numerical predictions into understandable behavioral insights, systematically answering the two core questions: which factors are the most important, and how do these factors specifically influence the decisions of different consumers? It provides a complete closed loop for the leap from data-driven to decision-supported.

Based on the prediction results of machine learning models and the analysis of SHAP explanations in the previous text, this paper further deepens the discussion of the mechanism of action of key variables from the perspective of consumer behavior theory. Specifically, several core variables revealed by the SHAP results show significant nonlinear marginal effects on consumers’ willingness to purchase ready-to-eat meals, indicating that consumers’ decisions do not follow the traditional linear utility maximization path but are more in line with the characteristics of bounded rationality and context dependence. For instance, when the perceived convenience and the level of safety trust cross a certain threshold, their promoting effect on purchase intention accelerates and increases. This is intrinsically consistent with the “risk perception inflection point effect” in prospect theory and the “critical activation mechanism” in perceived value theory. Meanwhile, the variables of price sensitivity and health cognition show alternating changes in inhibition and promotion in different intervals, reflecting consumers’ dynamic trade-offs among “cost-risk-utility”, and demonstrating obvious behavioral stratification characteristics.

Further combined with the SHAP interaction effect, it can be found that the consumer decision-making structure presents a hierarchical and nested decision-making logic: Food safety trust belongs to the basic constraint layer variable and has a “threshold filtering” effect on other perceived value variables; Convenience and taste perception constitute the experience-driven layer. Price and brand, on the other hand, fall into the rational trade-off level. This structural finding not only expands the explanatory boundaries of the traditional planned behavior theory (TPB) in the context of ready-to-eat meals, but also provides stratified intervention implications for food policy-making: that is, regulatory policies should prioritize strengthening the transparency of safety information and the construction of traceability systems to consolidate the foundation of trust; Industrial policies can enhance the value of experience through cold chain infrastructure and product innovation. At the market regulation level, demand activation can be achieved through price subsidies or brand certification. As a result, the significance of the model results has extended from merely predicting performance evaluations to the theoretical explanation level of consumer behavior mechanisms and policy intervention paths.

## 3. Results and Discussion

### 3.1. Descriptive Statistics and Data Reliability

Our survey shows that consumers in Jilin Province have a relatively high of level of acceptance of pre-prepared meals, with 70.0% (564 people) of the respondents indicating their willingness to purchase such products. These results indicate that the foundation of the pre-prepared meal market in this region is solid, which is in line with the trend of the expansion of this market across the country, driven by urbanization and changes in lifestyle. Reliability analysis confirmed that all scales had excellent internal consistency. The Cronbach α coefficient of the total table, consisting of 44 items, is 0.925, as shown in Table 1, far exceeding the excellent standard of 0.8, indicating that the overall structural design of the questionnaire is rigorous and the data quality is extremely high. The coefficients of all 10 dimensions exceed 0.86. The coefficients of the four dimensions of behavioral tendency (0.917), price promotion (0.914), risk safety (0.913), and convenience (0.902) have even surpassed the 0.9 mark, reaching an “excellent” level, indicating that consumers’ cognition and feedback in these core dimensions have extremely high consistency and stability and that the questionnaire items precisely measure the potential constructs. Compared with most research in this area, where some dimensions only reach the level of “0.7–0.8 (good)”, the data in this study are significantly more reliable.

The mean values of each dimension range from 3.669 to 3.787 (on a 5-point scale), indicating that the overall attitude of the respondents towards pre-prepared meals tended to be “relatively agreeable” or “positive”. The scores for purchase intention (3.787) and channel acquisition (3.782) were the highest, indicating that consumers in Jilin Province are very interested in purchasing pre-prepared meals and relatively satisfied with the convenience of the current purchase channels. The score for price promotion (3.669) was slightly lower, suggesting that consumers have some reservations about price sensitivity or that there is still room for improvement in promotional methods. The standard deviations of all dimensions are all around 1.1, indicating that the sample data not only reflect an overall positive trend but also maintain a reasonable degree of dispersion. This finding indicates that the surveyed groups are not completely homogeneous but rather have individual differences, a characteristic conducive to machine learning models’ ability to capture the characteristic patterns of different consumer groups.

In conclusion, the data from this survey exhibit extremely high reliability and good distribution characteristics. The design of the questionnaire items closely follows the psychology of consumers. Both emotional cognition and actual behavioral intentions are accurately quantified. These highly reliable data provide a solid and reliable empirical foundation for subsequent correlation analysis, Bayesian optimization modeling, and SHAP attribution.

### 3.2. Baseline Equilibrium Test and SMOTE–Tomek Data Balancing

Stratified random sampling was adopted in this study. The preprocessed valid samples were divided into a training set (n = 644) and a testing set (n = 161) in an 8:2 ratio. To verify the scientificity and unbiasedness of the division of the dataset and eliminate model evaluation bias caused by the differences in sample distribution, we constructed a Baseline Characteristics table. The Pearson chi-square test was used to statistically evaluate the distribution differences in the two groups of data in terms of target variables and demographic characteristics. The details of the result analysis are given below (see Table 2).

The distribution of the target variables is highly consistent. The distribution ratio of the “purchase intention (Target)” variable, which is at the core of the modeling, is extremely close between the training and the test sets. In the training set, the proportion of those “willing to purchase” was 70.0% (451 people), and the proportion of those “unwilling to purchase” was 30.0% (193 people). In the test set, 70.2% (113 people) were willing to purchase, while 29.8% (48 people) were unwilling to buy. The statistical test results show that the two groups of data have achieved a perfect balance for the target variable, and the stratified sampling strategy effectively guaranteed the homogeneity of the training and test sets.

In addition, there were no significant differences in demographic characteristics. The test results for the six key covariates—gender, age, education level, occupation, monthly income, and cooking frequency—showed that the *p*-values for all the variables were significantly greater than the statistical significance level of 0.05, indicating that the training and test sets originated from the same distribution of these characteristics.

The result of the gender difference test was *p* = 0.186, and the result of the age difference test was *p* = 0.365, indicating that the two groups of samples were evenly distributed in terms of basic population attributes. Notably, the proportion of young people aged 18 to 25 in the sample was relatively high (87.7% in the training set and 90.7% in the test set), which is in line with the younger nature of the market for pre-prepared meals. The statistical results for educational attainment (*p* = 0.145), occupation (*p* = 0.701), and monthly income (*p* = 0.399) all confirmed the balance of the two datasets in terms of socio-economic status. There was no significant difference in the distribution of cooking frequency, an important variable influencing purchasing decisions, between the two groups (*p* = 0.356), ensuring that the model would not overfit due to the distribution bias of living habits.

In conclusion, the results of the baseline equilibrium test (all *p* > 0.05) demonstrate the rationality and randomness of the division of datasets in this study. The training and test sets maintained a high degree of consistency in key features, providing a reliable data basis for the subsequent construction of machine learning models and ensuring that the results of the evaluation of the model on the test set truly reflect its generalization ability and are not a product of sample selection bias.

**Figure 1:**
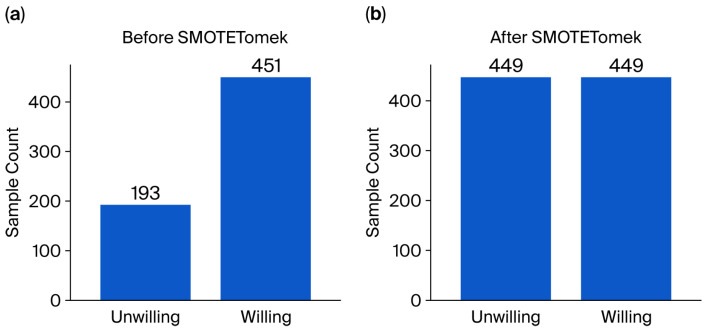
Class distribution of the training set before (**a**) and after (**b**) SMOTE–Tomek processing. Comparison of target variable labels (0: unwilling, 1: willing). (**a**) The original imbalanced distribution (2.3:1). (**b**) The balanced distribution achieved via the SMOTE–Tomek hybrid algorithm, which combines SMOTE oversampling for the minority class and Tomek Links for boundary cleaning to reduce noise and majority class bias.

Next, the training set was oversampled using the SMOTE–Tomek algorithm; the balance results are as follows:

The imbalance of the original distribution is shown in Figure 1a. In the original training set, the sample distribution exhibits a distinct “long tail” feature: the sample size of “willing to purchase (1)” is as high as 451 cases, while that of “unwilling to purchase (0)” is only 193 cases. This imbalance of approximately 2.3:1 can lead to a “majority class preference” in machine learning models during the training process. That is, in pursuit of overall accuracy, the model tends to directly determine ambiguous samples as “willing to purchase”, thereby seriously sacrificing the ability to identify “unwilling” groups.

The dual optimization of comprehensive sampling is shown in Figure 1b. After being processed by the SMOTE–Tomek algorithm, the training set achieved dual optimization of quantity balance and boundary cleaning.

Minority-Class Upsampling: The algorithm oversampled the minority classes that were “unwilling to purchase” and, through interpolation, synthesized a large number of new samples, causing the number to surge from 193 cases to 449 cases, leaving the model with sufficient data with which to determine the negative features that prevent consumers from making purchases (such as concerns about preservatives, price sensitivity, etc.).

Boundary Cleaning (Cleaning): Notably, the number of cases in which most classes were “willing to purchase” slightly decreased from the initial 451 to 449. This is evidence that the Tomek Links mechanism has taken effect: the algorithm identifies and eliminates two fuzzy samples for which there is extremely high confusion with other categories at the classification boundary.

Ultimately, the positive and negative samples were adjusted to a perfect balance of 449:449. This processing step not only eliminated the misleading influence of prior probability on the model but also created a clearer classification hyperplane by removing boundary noise, laying a data foundation for the subsequent improvement of the random forest model’s discrimination accuracy for “hesitant customers”.

### 3.3. Model Parameter Optimization and Performance Comparison

**Figure 2:**
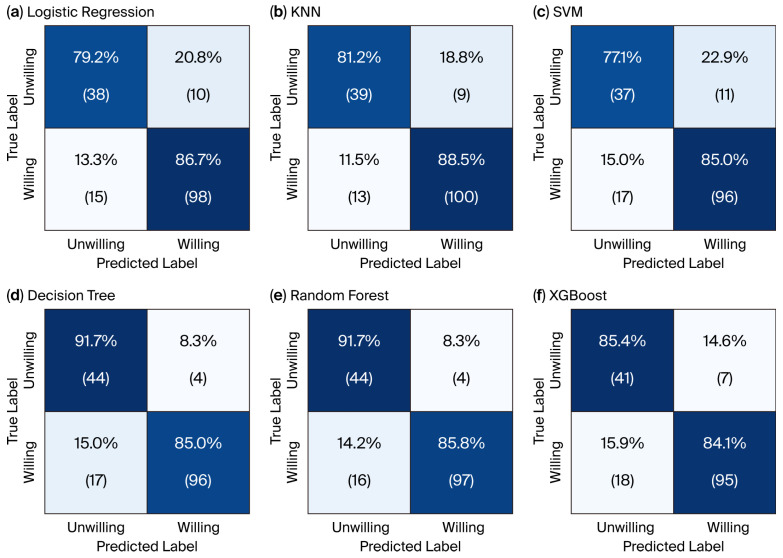
Confusion matrix heatmaps for the six classification models evaluated on the test set. Panels show (**a**) logistic regression, (**b**) KNN, (**c**) SVM, (**d**) decision trees, (**e**) random forest, and (**f**) XGBoost. Higher diagonal values indicate stronger agreement between predicted and actual consumer purchase intentions.

Subsequently, we conducted 80 rounds of Bayesian optimization on six models, with the F1 score serving as the optimization objective. The optimal parameters are shown in Table 3 below: 

After addressal of class imbalance and Bayesian hyperparameter optimization, the six models were evaluated by plotting their confusion matrix, as shown in Figure 2.

In the in-depth evaluation of model classification performance, random forest (RF) demonstrated a significant advantage in terms of true negative (TN) identification. As shown in Figure 2e, the model successfully identified 91.7% (44 cases) of samples with no purchase intent, achieving a false-positive rate (FPR) of merely 8.3%. This indicates that in practical applications, random forest can effectively mitigate the risk of misallocating marketing resources. By contrast, support vector machines (SVMs) and logistic regression (LR) exhibited false-positive rates as high as 22.9% and 20.8%, respectively, signifying greater marketing expenditure wastage. The high precision achieved by the random forest model stems primarily from its exceptionally low type I error rate, ensuring strong alignment between predictions and genuine potential customers. Regarding positive-sample capture, the random forest model attained a true-positive rate (TPR) of 85.8% (97 cases), demonstrating robust generalization capabilities while maintaining high precision.

When comparing the commercial decision boundaries across models, we found that KNN exhibited the lowest false-negative rate (11.5%). While this strategy maximizes the capture of potential customers, it comes at the cost of a high false-positive rate (18.8%), reflecting a trade-off between coverage and profitability. XGBoost delivers a more balanced overall performance, though it slightly trails random forest in terms of error rate control. Compared to the decision tree model in Figure 2d, random forest captures an additional high-value sample while maintaining the same low misclassification rate. This marginal gain demonstrates how ensemble learning algorithms effectively enhance model generalization by smoothing the classification bias inherent in individual decision trees.

As shown in Table 4, the random forest model demonstrated optimal comprehensive performance. It achieved the highest F1 score (0.907) and AUC value (0.928), indicating that it achieved the best balance between precision and recall rates and has the strongest discrimination ability. Critically, the precision rate of random forest is as high as 0.960, meaning that when it predicts a consumer is “willing”, the correct rate is 96%. This high predictive robustness and target specificity is of great value for enterprise application scenarios where parties are pursuing the return on marketing investment and striving to reduce the waste of resources for non-target customers. Therefore, we selected the random forest model for in-depth explanation.

This model perfectly meets enterprises’ chief demand for “cost reduction and efficiency improvement.” It can not only ensure the reliability of the overall prediction through a high AUC but also guarantee that every marketing budget is spent where it is most needed through a high accuracy rate of 0.876. As an ensemble tree model based on bagging, the random forest naturally adapts to the subsequent MDI feature importance analysis and SHAP attribution framework. Therefore, the subsequent feature importance analysis and SHAP interpretability research were carried out based on the random forest model.

### 3.4. The Hierarchical Structure and Attribution Analysis of Core Drivers of Purchase Intention

**Figure 3:**
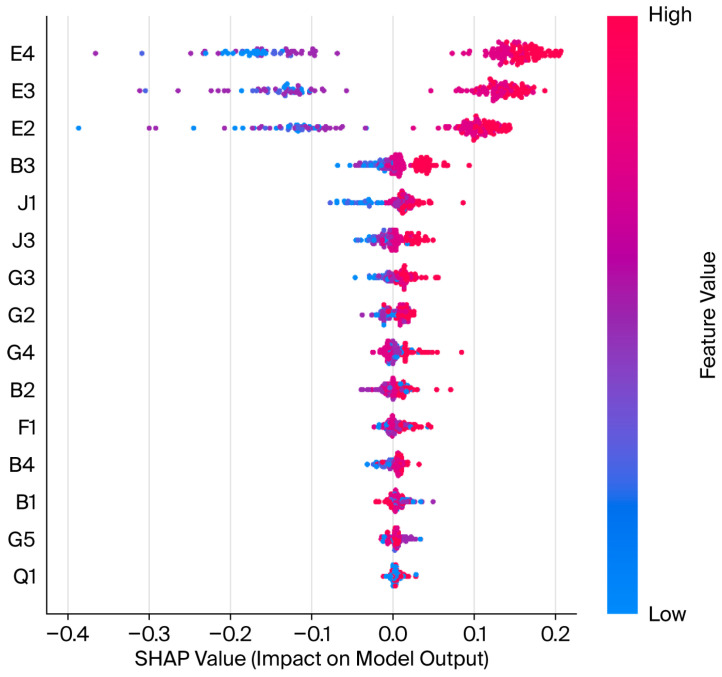
SHAP bee swarm plot illustrating the impact of features on consumer purchase intention with respect to the random forest model. Features are ranked by their global importance. Each dot represents a single instance. The horizontal axis (SHAP value) indicates whether the feature increases (positive) or decreases (negative) the likelihood of purchase. Color represents the feature value (red, high; blue, low). For example, high “Recommendation Willingness (E4)” (red dots on the right) strongly promotes purchase intention.

**Figure 4:**
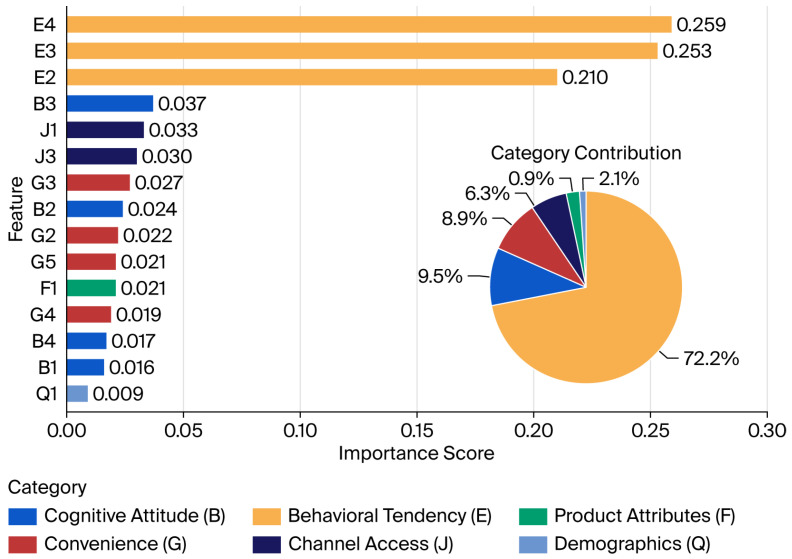
Global feature importance ranking based on Mean Decrease Impurity (MDI) and cumulative contribution by category. This figure illustrates the quantitative contribution of 15 screened predictor variables to the random forest (RF) model. The bar chart (left) displays the importance scores calculated via the MDI algorithm, which measures the average reduction in Gini impurity across all decision trees. “Recommendation Willingness (E4)” emerged as the most critical determinant, with an importance score of 0.259. The pie/cumulative chart (right) aggregates individual features into four psychological and environmental dimensions. The dimension behavioral tendency (E) demonstrates overwhelming explanatory power, accounting for 72.2% of the total predictive contribution, while cognitive attitude (B), convenience (G), and channel access (J) provide the rational foundation for consumer decisions.

We integrated random forest feature importance (MDI) with a SHAP bee swarm plot to construct a ‘hierarchical-driven attribution model’ for pre-prepared meal purchase intent (see Figure 3 and Figure 4). The results indicate that consumer decision-making logic exhibits distinct hierarchical characteristics: ‘subjectively intention-driven, rationally cognitively supported, and weakly influenced by attributes’.

Our research indicates that the dimension behavioral tendency (E) accounts for a cumulative contribution rate of 72.2% with respect to model predictions, constituting the absolute core of the decision-making mechanism. Among the dimensions, recommendation willingness (E4) ranks first, with an importance score of 0.259. The SHAP bee swarm plot reveals an extreme ‘red–right, blue–left’ distribution for E4. The high-score samples (red) exhibit marginal contributions that significantly elevate prediction probabilities, peaking near 1.5, while low-score samples (blue) cluster predominantly in negative values. This finding confirms recommendation willingness has a veto effect within the decision chain, serving as the core threshold for gauging deep trust and purchase conversion. Furthermore, “Prioritize” (E3) and “Increase Frequency” (E2) form a robust positive driving force. The high compactness of E3’s high-score grid reflects the strong driving role of psychological predisposition in final transactions, while E2’s widespread distribution in the negative zone reveals that the lack of incremental purchase plans is the primary risk factor leading to potential customer attrition.

Cognitive attitudes (B, 9.5%), convenience (G, 8.9%), and channel accessibility (J, 6.3%) form the rational foundation of decision-making. Diverse selection (B3) leads among non-preference-based indicators, demonstrating that a rich product portfolio effectively strengthens consumers’ value recognition. Concurrently, the inclusion of channel convenience (J1) and after-sales policies (J3) indicates that robust supply chain arrangements and low trial-and-error costs are pivotal for eliminating purchase hesitation and completing the decision-making loop. Functional attributes such as ease of storage (G3), though exhibiting low dispersion in the SHAP plot, serve as foundational thresholds providing sustained underlying support for consumption decisions.

The cumulative contribution rate for product attributes (F) and demographic characteristics (Q) falls below 4%. Notably, traditional sensory evaluation metrics like flavor (F1) rank lower, with SHAP values fluctuating narrowly near the zero axis. This suggests that in the current market environment, consumers demonstrate considerable tolerance of flavor nuances once purchase intent and convenience requirements are met. Furthermore, the SHAP plots for variables like gender (Q1) cluster tightly around the centerline with minimal dispersion, confirming that pre-prepared meal consumption has achieved cross-group universality within the study region. Purchase decisions are more driven by individual subjective psychology than they are constrained by physiological attributes or socio-demographic characteristics.

### 3.5. Nonlinear Effects of Key Features and the Deconstruction of Individual Decision-Making Mechanisms

**Figure 5:**
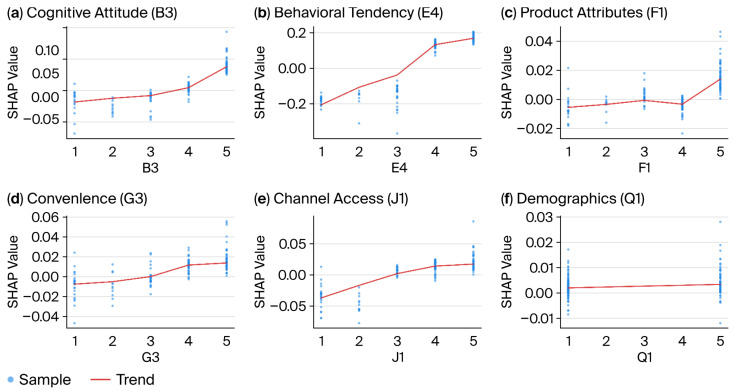
SHAP dependency plots revealing nonlinear threshold effects of key drivers. These plots show the marginal effect of a single feature on the predicted outcome: (**a**) cognitive attitude (B3); (**b**) behavioral tendency (E4); (**c**) product attributes (F1); (**d**) convenience (G3); (**e**) channel access (J1); and (**f**) demographics (Q1). The S-shaped curve for E4 (recommendation willingness) reveals a critical threshold at 3 points, where purchase intention undergoes a qualitative shift.

**Figure 6:**
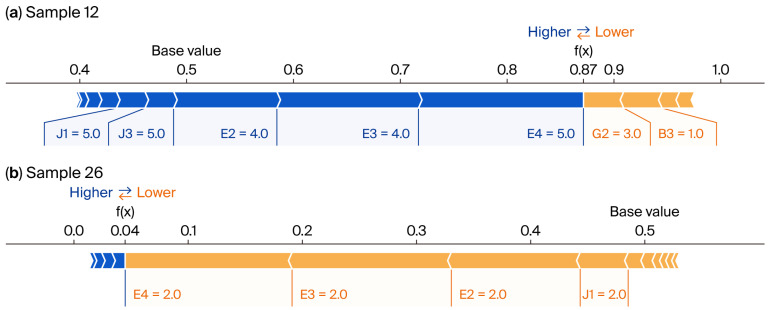
SHAP force plots deconstructing the decision-making logic of individual samples, showing predictions for (**a**) a high-willingness customer and (**b**) a low-willingness customer. Red arrows indicate features pushing the prediction higher (positive impact), while blue arrows represent features pushing the prediction lower (negative impact). The base value represents the average model output across all samples.

Through coupled analysis of SHAP dependency plots (Figure 5) and individual sample force plots (Figure 6), this study reveals, at the micro level, the nonlinear threshold effects and asymmetric game mechanisms underlying pre-prepared meal consumption decisions.

E4 exhibits a pronounced S-shaped curve. When scores fall within the inhibition zone (1–3 points), SHAP values are negative (approximately −0.2 to −0.05), significantly suppressing purchasing behavior. However, within the 3–4-point range, the curve steeply increases, with SHAP values rapidly turning positive. This indicates that 3 points represents a critical “trust threshold”, where purchase conversion undergoes qualitative change only when consumer attitudes shift from “neutral” to “willing to recommend”. Both the cognitive dimension (B3) and channel/convenience dimensions (J1 and G3) exhibit positive linear pull effects. Specifically, B3 (diverse selection) exhibits a robust positive correlation with purchase probability, indicating that SKU richness has a sustained cumulative effect. J1 (channel convenience) demonstrates standard linear growth, validating the ‘channel is king’ logic in directly lowering physical barriers and boosting sales. Notably, F1 (flavor freshness) exhibits non-monotonic behavior. Within the 1–4-point range, its influence on decision-making is weak (SHAP values approach zero); only at the 5-point level (strongly agree) does the SHAP value significantly surge (>0.02). This finding reveals the ‘high-demand nature’ of the food safety dimension, where only an exceptional experience can dispel concerns and translate into a purchasing incentive. The fitted line for Q1 (gender) is nearly horizontal and closely aligned with the 0-axis, a micro-level confirmation that the variable gender exhibits no differentiation in pre-cooked meal consumption decisions. This consumption pattern has transcended gender divides to become a universal choice.

By comparing extreme samples (Figure 6), this study reveals decision-making asymmetry. High-willingness sample (Sample 12, f(x) = 0.87)—long-board compensation mechanism: This respondent expressed strong dissatisfaction with product variety (B3 = 1.0), yet external service dividends successfully compensated for this single product attribute deficiency. This effect was driven by the synergistic effects of extreme channel convenience (J1 = 5.0), comprehensive after-sales support (J3 = 5.0), and a strong intrinsic desire to recommend (E4 = 5.0). This indicates purchasing decisions follow an ‘additive principle’, where synergistic reinforcement of multi-dimensional strengths enables transaction completion. Low-willingness sample (Sample 26, f(x) = 0.04)—collapse effect was observed due to a lack of willingness: All of this respondent’s core metrics (E2, E3, and E4) remained low (2.0), with effort nearly entirely obscured by the blue resistance band. Even with potential convenience incentives present, the comprehensive absence of subjective motivation constitutes an insurmountable cognitive barrier. This indicates rejection decisions follow a ‘multiplicative principle’, where the absence of a single core element (such as intrinsic willingness falling below a negative threshold) causes the purchase probability to drop exponentially to zero.

## 4. Conclusions

We constructed a decision analysis framework for pre-prepared meal consumption intent by integrating rigorous statistical validation with advanced machine learning algorithms. Among the six machine learning algorithms evaluated, the random forest model demonstrated the most outstanding performance in predicting consumer purchase intent, achieving an F1 score of 0.9065 and a precision rate as high as 0.9604. This high precision, coupled with an extremely low misclassification rate, provides a robust digital technology foundation for enterprises transitioning from extensive traffic acquisition to precise marketing-based retention operations. Consumer decision-making exhibits a distinct hierarchical structure centered on intention and grounded in rationality. Feature importance (MDI) and SHAP global summarization analysis indicated that behavioral inclination (Dimension E), particularly recommendation willingness (E4, with an importance score of 0.259), is the key driver of purchasing behavior. Rational factors such as channel convenience (J1) and choice diversity (B3) serve as essential supporting pillars. SHAP dependency graph analysis identified a pronounced S-shaped trust threshold effect for recommendation willingness. Purchase probability undergoes a qualitative leap only after attitude scores surpass the neutral threshold. Furthermore, individual sample power diagrams were used to deconstruct the asymmetric logic of decision-making: purchase decisions follow a compensatory mechanism (where service benefits offset product shortcomings), while rejection decisions adhere to a non-compensatory mechanism (where the absence of core willingness constitutes a veto). Crucially, these machine learning outputs facilitate a novel theoretical synthesis that extends traditional behavioral frameworks. While the Theory of Planned Behavior (TPB) correctly identifies intention as a precursor to action, our SHAP-based conceptual model reveals that this relationship is nonlinear and subject to critical ‘activation thresholds’ (e.g., the S-shaped trust threshold). Furthermore, the observed asymmetric compensatory logic empirically advances Prospect Theory within the food context: negative perceptions (e.g., lack of core willingness) function as non-compensatory loss domains that drastically collapse purchase probability, whereas positive rational enablers (e.g., channel convenience) operate in an additive gain domain. This synthesized framework bridges the gap between predictive algorithms and bounded rationality, offering a more nuanced behavioral model for food consumption. These findings provide empirical evidence for optimizing supply chains in the pre-prepared meal industry and suggest that regulatory authorities should prioritize risk perception and information transparency to establish a trust-based social governance system.

## 5. Limitation and Generalizability

Although this paper strives for rigor in model construction and empirical analysis, the research is still inevitably limited by the range of regional samples. The data of this study mainly come from Jilin Province. This region has certain geographical context particularities in terms of the development stage of the ready-to-eat food industry, the foundation of cold chain logistics, the dietary structure of residents and the level of risk awareness. For instance, consumers in Northeast China generally have a higher acceptance of frozen and pre-processed foods, and are more sensitive to food safety and storage conditions. This may, to a certain extent, enhance the explanatory power of the variables of safety trust and convenience perception. Furthermore, the demographic structure of the sample exhibits a significant skew, with over 87% of respondents aged 18–25. While this accurately reflects the core consumer base driving the current pre-prepared meal market—a demographic characterized by fast-paced urban lifestyles and a high acceptance of convenience foods—it inevitably limits the external validity of our findings regarding older populations. The decision-making logic of older demographics may fundamentally differ, potentially prioritizing nutritional attributes, traditional cooking habits, and health cognitions over convenience. Therefore, extrapolating the current model’s hierarchical structure to a generalized, all-age population requires caution. Therefore, the weights of relevant variables and their nonlinear threshold intervals may have the embeddedness characteristics of regional consumption culture and industrial structure.

Based on this, the research conclusions of this paper should be extrapolated to other regional or national contexts with caution. For first-tier cities with highly mature ready-to-eat food industries or developed countries with more complete cold chain systems, consumers’ decision-making structures may place more emphasis on brand and nutritional attributes. In regions at the early stage of industrial development, price and availability may become the dominant factors. Future research can test the stability and generalizability of the model structure and variable effects in this paper through cross-regional comparative samples, international multi-center data or longitudinal follow-up surveys, thereby further enhancing the external validity and policy applicability of the research conclusions.

## Figures and Tables

**Table 1:** Results of the reliability analysis of the consumer survey scale for pre-prepared meals. This table presents the internal consistency of the multi-dimensional scale based on 805 valid samples. It includes the number of items, mean scores, and standard deviations (SDs) for each psychological and behavioral construct. Cronbach’s α coefficients are reported for each dimension (e.g., emotional attitude and behavioral tendency) to verify measurement stability, with values > 0.9 indicating excellent reliability.

Dimension	Number	Mean	SD	Cronbach’s α
Emotional attitude	4	3.723	1.104	0.883
Cognitive attitude	4	3.731	1.100	0.882
Subjective norm	4	3.741	1.078	0.871
Perceptual control	4	3.720	1.100	0.880
Purchase intention	4	3.787	1.052	0.860
Behavioral tendency	5	3.715	1.139	0.917
Convenience	5	3.690	1.099	0.902
Risk safety	5	3.703	1.137	0.913
Price promotion	5	3.669	1.131	0.914
Channel acquisition	4	3.782	1.087	0.879
Overall scale	44	3.723	0.628	0.925

**Table 2:** Baseline equilibrium test and demographic distribution between the training and testing sets. Comparative analysis of demographic variables (gender, age, education, etc.) and the target variable (purchase intention) between the training (*n* = 644) and testing (*n* = 161) datasets. Pearson’s chi-square χ^2^ test was used to calculate *p*-values, where *p* > 0.05 indicates a balanced distribution, ensuring that the model evaluation reflects the true patterns without selection bias.

Variables	Training Set (*n* = 644)	Testing Set (*n* = 161)	Statistical Value	*p*-Value
Target			χ^2^ = 0.000	1.000
Unwilling	193 (30.0%)	48 (29.8%)		
Willing	451 (70.0%)	113 (70.2%)		
Gender			χ^2^ = 1.752	0.186
Female	332 (51.6%)	93 (57.8%)		
Male	312 (48.4%)	68 (42.2%)		
Age			χ^2^ = 0.820	0.365
18–25	565 (87.7%)	146 (90.7%)		
Over 56	79 (12.3%)	15 (9.3%)		
Education			χ^2^ = 5.404	0.145
High school and below	147 (22.8%)	41 (25.5%)		
Junior college	157 (24.4%)	50 (31.1%)		
Undergraduate	241 (37.4%)	53 (32.9%)		
Master’s degree or above	99 (15.4%)	17 (10.6%)		
Occupation			χ^2^ = 3.820	0.701
Other	95 (14.8%)	23 (14.3%)		
Student	69 (10.7%)	18 (11.2%)		
Enterprise employee	190 (29.5%)	47 (29.2%)		
Civil servant	78 (12.1%)	17 (10.6%)		
Professional	111 (17.2%)	28 (17.4%)		
Sole proprietorship	46 (7.1%)	18 (11.2%)		
Retirement	55 (8.5%)	10 (6.2%)		
Income			χ^2^ = 4.054	0.399
Below CNY 3000	140 (21.7%)	43 (26.7%)		
CNY 3000–6000	173 (26.9%)	35 (21.7%)		
CNY 6000–10,000	176 (27.3%)	50 (31.1%)		
CNY 10,000–15,000	107 (16.6%)	23 (14.3%)		
Over CNY 15,000	48 (7.5%)	10 (6.2%)		
Cooking Frequency			χ^2^ = 4.392	0.356
Almost never	43 (6.7%)	7 (4.3%)		
Very infrequently	80 (12.4%)	20 (12.4%)		
1–2 times a week	97 (15.1%)	28 (17.4%)		
3–5 times a week	166 (25.8%)	51 (31.7%)		
Every day	258 (40.1%)	55 (34.2%)		

**Table 3:** Optimal hyperparameters for machine learning models determined via Gaussian process-based Bayesian optimization.

Model	Optimal Parameters
LR	C = 0.41, penalty = 11, solver = liblinear
KNN	n_neighbors = 4, weights = uniform, *p* = 1
SVM	C = 2.22, kernel = rbf, gamma = scale
DT	max_depth = 10, min_samples_split = 9, criterion = gini
RF	n_estimators = 145, max_depth = 19, bootstrap = True
XGB	n_estimators = 59, learning_rate = 0.17, max_depth = 3

**Table 4:** Performance evaluation metrics for the six machine learning classification models on the independent test set. The metrics include F1 score, accuracy, recall, precision, and the area under the curve (AUC). The random forest (RF) model achieved the highest F1-score (0.907), demonstrating its superior ability to handle heterogeneous consumer data.

Model	F1 Score	Accuracy	Recall	Precision	AUC
LR	0.887	0.845	0.867	0.907	0.900
KNN	0.901	0.863	0.885	0.917	0.882
SVM	0.873	0.826	0.850	0.897	0.885
DT	0.901	0.870	0.850	0.960	0.917
RF	0.907	0.876	0.858	0.960	0.928
XGB	0.884	0.845	0.841	0.931	0.921

## Data Availability

The original contributions presented in this study are included in the article/Supplementary Materials. Further inquiries can be directed to the corresponding author.

## Supplementary Materials

The following supporting information can be downloaded at: https://www.mdpi.com/article/10.3390/foods15050896/s1, Figure S1: ROC curve comparison chart, Figure S2: SHAP global heat map, Figure S3: Heat map of the correlation coefficient between the TOP15 features and the target variable, Table S1: Table of abbreviations and the meanings of questionnaire variables, Table S2: Summary of the coding rules for scale questions and categorical variables.
