# Supplementary material for: Predicting Consumer Purchase Intention for Pre-Prepared Meals Based on Random Forest and Explainable AI (SHAP): A Study in Jilin Province, China
Source: Foods. 2026 Mar 5;15(5):896. doi: 10.3390/foods15050896 (PMC12984385; doi:10.3390/foods15050896)
Supplement: Supplementary file 1 [file foods-15-00896-s001.zip › foods-4154126-supplementary.pdf]

**Supplementary Materials:**

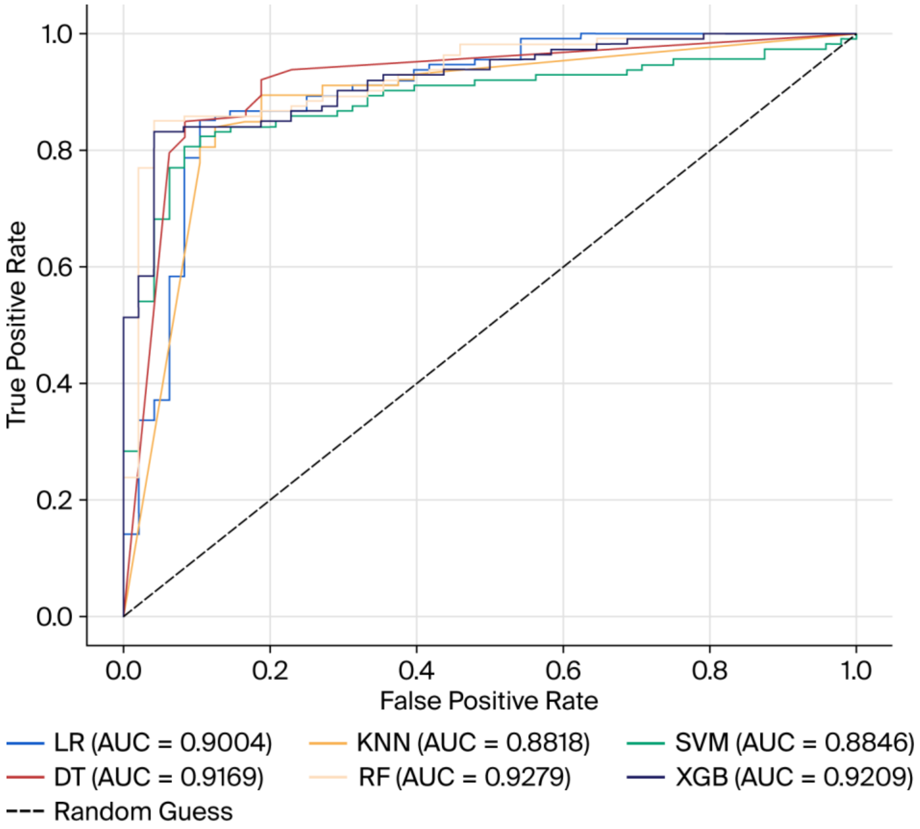


**Figure S1:** ROC curve comparison chart. The ROC curve of random forest presents the most full "upper-left convex" shape, and its area under the curve (AUC) reaches 0.9279. Thus, when the model randomly selects a sample of people "willing to purchase" and a sample of people "unwilling to purchase", the probability that it can provide a correct ranking (i.e., that the probability of positive samples is greater than that of negative samples) is close to 93%. This result indicates that random forest not only significantly outperforms random guessing (AUC =0.5) but also narrowly defeats XGBoost (AUC = 0.9209), establishing its position as the model with the strongest discriminative ability in this study.

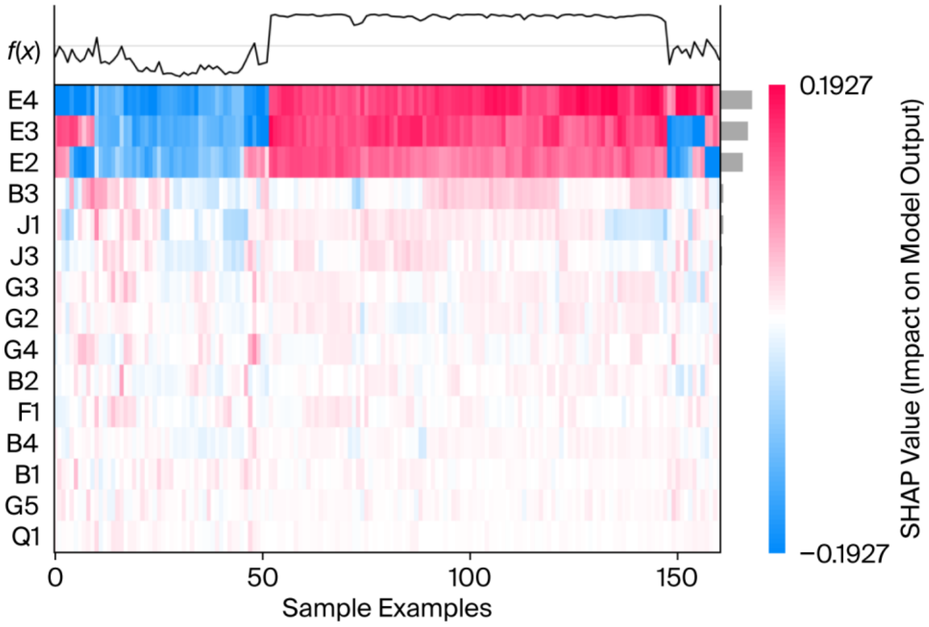

**Figure S2:** SHAP global heat map. The global prediction attribution matrix of the random forest model for the test-set samples is presented. The distinct "red and blue boundary" in the figure reveals that the consumer group of pre-prepared meals has two completely different decision-making patterns, and this difference is mainly dominated by behavioral tendency (E-dimension) characteristics.

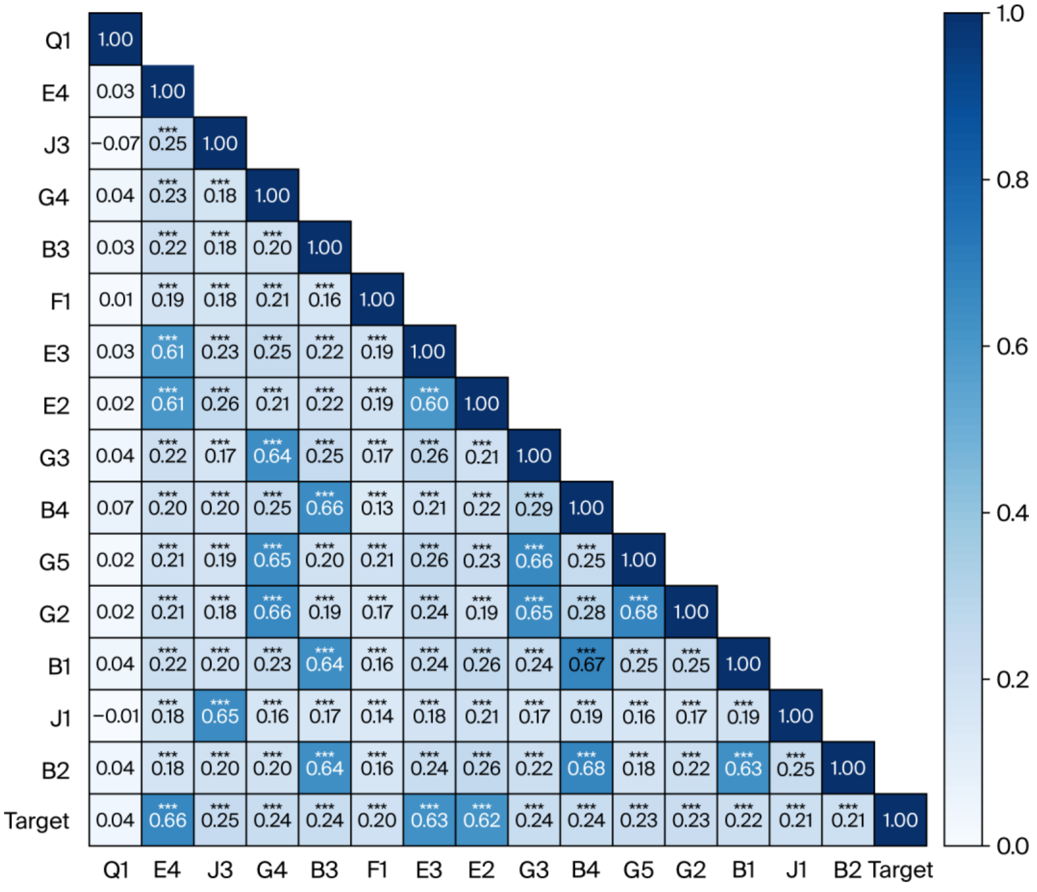


**Figure S3.** Heat map of the correlation coefficient between the TOP15 features and the target variable. The Pearson correlation coefficient was used to measure the linear association strength between each feature and the target variable (target, purchase intention), and the significance test was conducted in combination with the two-tailed t-test. As shown in the figure, the heat map not only presents the magnitude of the correlation coefficient (color depth) but also marks the statistical significance level with an asterisk ($p < 0.001$). Values represent Pearson correlation coefficients. Statistical significance: *** $p < 0.001$; ** $p < 0.01$; * $p < 0.05$.

**Table S1.** Table of abbreviations and the meanings of questionnaire variables.

| Variable Abbreviation | Questionnaire Dimensions | Variable Meaning and Item Content | Types of Variables |
|---|---|---|---|
| A1–A4 | Emotional attitude | Emotional experiences at the time of purchase (e.g., convenience and ease, pleasantness, the feeling of making a wise choice, and efficiency) | Continuous (scale) |
| B1–B4 | Cognitive attitude | Rational cognitive assessment (such as saving cooking time, reducing the trouble of preparing dishes, diversifying one's diet, and the notion that the overall benefits outweigh the disadvantages) | Continuous (scale) |
| C1–C4 | Subjective norm | Social environment influence (such as family recognition, recommendations from significant others, recommendations on social media platforms, and purchases by peers) | Continuous (scale) |

| | | | |
|---|---|---|---|
| D1–D4 | Perceptual control | Self-assessment of abilities (such as purchasing convenience, knowledge selection, cooking skills, and decision-making control) | Continuous (scale) |
| E1 | Willingness to buy | The degree to which one is willing to purchase pre-prepared meals in the coming month (source of the target variable) | Dichotomy |
| E2–E4 | Behavioral disposition | Increases or decreases in purchase frequency plans, willingness to buy, and willingness to recommend to others | Continuous (scale) |
| F1–F5 | Product attributes | The extent to which food freshness, nutritional components, taste and flavor, packaging information, and brand reputation have an influence | Continuous (scale) |
| G1–G5 | Convenient and practical | The extent to which a product saves cooking time, simplifies steps, is easy to store, reduces waste, and has an impact on emergency backup value | Continuous (scale) |
| H1–H5 | Risk safety | The degree of concern over additives, production hygiene, risk of deterioration, inconsistency in publicity, and uncertainty of health impacts | Continuous (scale) |
| I1–I5 | Price promotion | The extent to which price rationality, comparison of takeout prices, attractiveness of discounts, membership benefits, and the cost-effectiveness of set meals have an influence | Continuous (scale) |
| J1–J4 | Channel acquisition | The extent to which the convenience of purchase channels, delivery speed, return and exchange policies, and the quality of customer service have an influence | Continuous (scale) |
| P1 | Purchasing behavior | The average monthly frequency of purchasing pre-prepared meals | Ordered categories |
| P2 | Purchasing behavior | The usual amount for a single purchase of pre-prepared meals | Ordered categories |
| Q1 | Demographic statistics | Gender | Unordered categories |
| Q2 | Demographic statistics | Age | Ordered categories |
| Q3 | Demographic statistics | Education background | Ordered categories |
| Q4 | Demographic statistics | Occupation | Unordered categories |
| Q5 | Demographic statistics | Monthly income | Ordered categories |
| Q6–Q7 | Demographic statistics | Residence and family structure | Unordered categories |
| Q8 | Demographic statistics | Cooking frequency | Ordered categories |
| Target | Willingness to buy | Purchase intention for pre-prepared meals (derived from the E1 code value) | Binary classification (target variable) |

**Table S2.** Summary of the coding rules for scale questions and categorical variables.

| Types of Variables | Original Text → Coded Value |
|---|---|
| Scale questions (A–J) | ‘Strongly disagree’, ‘Zero impact’, ‘Know nothing’ → 1;<br>‘Don’t quite agree’, ‘Less effect’, ‘Heard but don’t understand’ → 2;<br>‘General’, ‘General Influence’, ‘Broadly understood’ → 3;<br>‘Relatively agree’, ‘Have a great influence’, ‘Acquaint entirely’ → 4;<br>‘Totally consent’, ‘Very influential’ → 5;<br>‘(Skip), (Empty)’ → 0 |
| Ordered categories (P1) | ‘Less than once’ → 1;<br>‘1–2 times’ → 2;<br>‘3–4 times’ → 3;<br>‘5–6 times’ → 4;<br>‘More than 7 times’ → 5;<br>‘(Skip)’ → 0 |
| Ordered categories (P2) | ‘Less than 50 CNY’ → 1;<br>‘51–100 CNY’ → 2;<br>‘101–200 CNY’ → 3;<br>‘201–300 CNY’ → 4;<br>‘More than 300 CNY’ → 5 |
| Ordered categories (Q2) | ‘Age 18–25’ → 1;<br>‘Age 26–35’ → 2;<br>‘Age 36–45’ → 3; |

| | |
|---|---|
| | 'Age 46–55' → 4;<br>'Over age 55' → 5 |
| Ordered categories (Q3) | 'High school and below' → 1;<br>'Junior college' → 2;<br>'Regular college' → 3;<br>'Master's degree or above' → 4 |
| Ordered categories (Q5) | 'Less than 3000 CNY' → 1;<br>'3000–6000 CNY' → 2;<br>'6000–10,000 CNY' → 3;<br>'10,000–15,000 CNY' → 4;<br>'More than 15,000 CNY' → 5 |
| Ordered categories (Q8) | 'Hardly cook' → 1;<br>'Seldom cook' → 2;<br>'1–2 times a week' → 3;<br>'3–5 times a week' → 4;<br>'Cook almost every day' → 5 |
| Unordered categories (Q1) | 'Female' → 0;<br>'Male' → 1 |
| Unordered categories (Q4) | 'Professional' → 0;<br>'Self-employed individuals/entrepreneurs' → 1;<br>'Employees of the enterprises' → 2;<br>'Civil servants/Personnel of public institutions' → 3;<br>'Student' → 4;<br>'Freelancer' → 5;<br>'Retiree' → 6 |
| Unordered categories (Q6) | 'Jilin' → 0;<br>'Siping' → 1;<br>'Yanbian' → 2;<br>'Songyuan' → 3;<br>'Baicheng' → 4;<br>'Baishan' → 5;<br>'Liaoyuan' → 6;<br>'Tonghua' → 7;<br>'Changchun' → 8 |
| Unordered categories (Q7) | 'Of three generations under one roof' → 0;<br>'Live with parents' → 1;<br>'Live in solitude' → 2;<br>'A couple' → 3;<br>'Families with minor children' → 4 |
| Target variable | E1 ∈ [4, 5] → 1 (Be willing to);<br>E1 ∈ [1, 2, 3] → 0 (Unwillingness) |
